# Research on the Design of an Optical Information Storage Sensing System Using a Diffractive Optical Element

**DOI:** 10.3390/s131115409

**Published:** 2013-11-08

**Authors:** Xuemin Cheng, Qun Hao, Jianbo Hou, Xiangping Li, Jianshe Ma, Min Gu

**Affiliations:** 1 Graduate School at Shenzhen, Tsinghua University, Shenzhen 518055, China; E-Mails: houjb10@mails.tsinghua.edu.cn (J.H.); mjs888@126.com (J.M.); 2 Shenzhen Key Laboratory of LED Packaging, Shenzhen 518055, China; 3 School of Optoelectronics, Beijing Institute of Technology, Beijing 100081, China; E-Mail: qhao@bit.edu.cn; 4 Centre for Micro-Photonics, Faculty of Engineering and Industrial Sciences, Swinburne University of Technology, Hawthorn, Victoria 3122, Australia; E-Mails: xiangpingli@swin.edu.au (X.L.); mgu@swin.edu.au (M.G.)

**Keywords:** optical information storage, sensing system, gold nanorods, diffractive optical element, three-dimensional optical data storage, PACS 42.79.Pw

## Abstract

This paper introduces a compact optical information storage sensing system. Applications of this system include longitudinal surface plasmon resonance detection of gold nanorods with a single femtosecond laser in three-dimensional space as well as data storage. A diffractive optical element (DOE) is applied in the system to separate the recording-reading beam from the servo beam. This allows us to apply a single laser and one objective lens in a single optical path for the servo beam and the recording-reading beam. The optical system has a linear region of 8 λ, which is compatible with current DVD servo modules. The wavefront error of the optical system is below 0.03 λ_rms_. The minimum grating period of the DOE is 13.4 μm, and the depth of the DOE is 1.2 μm, which makes fabrication of it possible. The DOE is also designed to conveniently control the layer-selection process, as there is a linear correlation between the displacement of the DOE and the layer-selection distance. The displacement of DOE is in the range of 0–6.045 mm when the thickness of the layer-selection is 0.3 mm. Experiments were performed and the results have been verified.

## 1. Introduction

An optical information sensor can be applied in many fields, such as optical storage and lightshows, as the quantities of the beam in the system can be detected, delivering macroscopic observation of the microscopic properties of a material [1]. Gold nanorods are one type of nanomaterial. They have the unique property of longitudinal surface plasmon resonance (LSPR) [2–8], which has a good sensitivity of wavelength and polarization. When the LSPR effect occurs, a large amount of luminous energy is absorbed by the gold nanorods, and two-photon fluorescence is generated.

Exploiting the wavelength and polarization sensitivity of the LSPR effect of gold nanorods, Gu proposed true five-dimensional optical recording [9], including three spatial dimensions, wavelength, and polarization. In the recording process, data is recorded by the photothermal recording mechanism. In the reading process, gold nanorods are induced to generate two-photon fluorescence. In the following static experiment using DVD compatible apparatus [10], the potential of ultra-high density, three-dimensional optical memory of dual-layer recording in gold-nanorod-dispersed discs with an equivalent capacity of 69 GB was demonstrated.

In this paper, upon the previous research work on gold-nanorod-dispersed discs, we propose a novel optical information sensor configuration which consists of a single-laser opto-electronic system applied to a specified multilayer disc structure. The multilayer disc structure in the sensor is designed with the groove structure on the substrate as the servo layer, the recording layers and spacer layers alternately coated on the substrate. The servo beam and recording-reading beam are required to independently focus on different layers in the multi-dimensional space, which is integrated in a single compact optical path with one laser and one objective lens by the use of one DOE. The corresponding reading and recording characteristics of the sensor are designed to be compatible with the standard DVD platform. Some applications of the proposed system could also be optical information sensing in micromachining, sensors, optical encryption and more as well as multi-dimensional optical data storage considering the LSPR effect of gold nanorods.

## 2. Basic Configuration

### 2.1. Scheme

**Figure 1.**
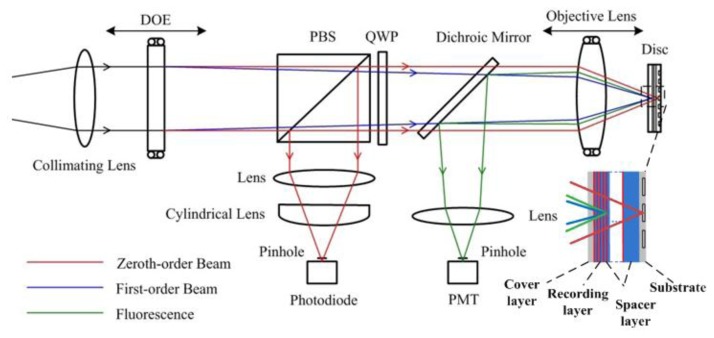
Configuration of the optical information storage sensing system.

In the current scheme of the sensor, a laser beam is delivered to the optical path, passes through the DOE, a polarized beam splitter (PBS), a quarter-wave plate (QWP), a dichroic mirror, and the objective lens sequentially; then the zeroth-order and first-order beams focus on the multilayer disc as the servo beam and the recording-reading beam respectively, as shown in Figure 1.

The specified disc structure is also shown in Figure 1. Considering the crosstalk [11], the depth of spacer layer between the adjacent recording layers is set to 5 μm. According to the present level of technology, the thicknesses of substrate and cover layer are 0.1 mm and recording layer to 1 μm so that the 50-layer cascaded storage layers can be realized in the 300 μm medium. Thus the multilayer disc is designed to achieve the total capacity of 235 GB in the three-dimensional space with each layer containing 4.7 GB of data when adopting a similar structure as a DVD-RW.

To be compatible with the multilayer disc structure, the DOE is designed so that the zeroth-order beam focuses on the servo layer for focusing and tracking, while the first-order diffraction beams focuses on the recording layer. The two beams are controlled and focus on the corresponding layers independently when changing the positions of the objective lens and the DOE. Therefore, the zeroth-order beam retains a constant focus position on the disc substrate, regardless of any DOE position change as the optical power of the DOE is zero [12]; and then the beam is focused on the substrate by the objective lens as the focus position is changed with the movement of the object lens. It is reflected and passes through the objective lens, the dichroic mirror, and the QWP. The polarization status of the beam changes as it passes through the QWP twice and is reflected by the PBS; thereby, the servo beam is separated from the main optical path and focused on a photodiode for the detection of disc-focusing and disc-tracking error signals. The first-order beam is used as the recording-reading beam, controlled by the DOE movement to focus on the corresponding storage layer where fluorescence occurs. The excited fluorescence is collected by the objective lens and extracted from the main optical path by the dichroic mirror and detected by a photomultiplier tube (PMT). The remaining recording beam is filtered out by the color filter. In this system, two beams are realized in a single main optical path by the use of one laser and one objective lens, which might prove to be a reliable opto-electronic system with fewer sources of errors. It is also possible for the servo module to function in this role as it is compatible with that used in the current commercial optical disc system.

### 2.2. Specifications

**Figure 2.**
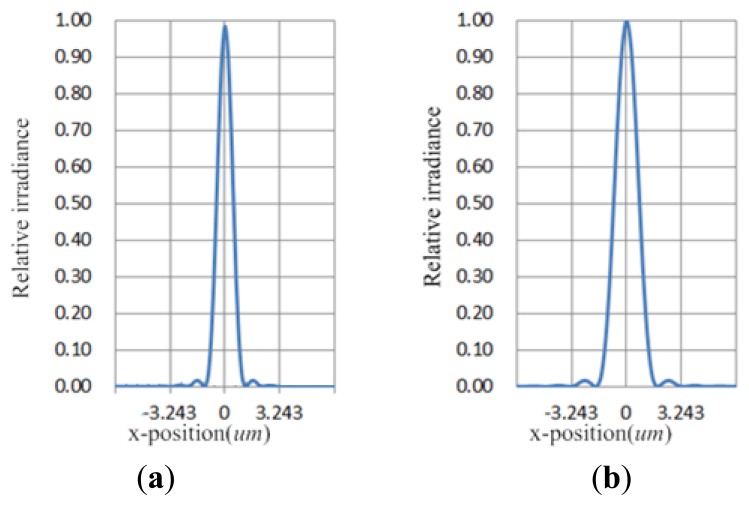
PSF cross section of (**a**) the zeroth-order beam and (**b**) the first-order beam.

The optical model of this system was built in Zemax 2010 (Radiant Zemax, Redman, WA, USA) by a wavelength of 780 nm and an objective of NA = 0.6 [10]. The wavefront error of the optical system is below 0.03 λ_rms_. The DOE is designed using the phase function. As the fluorescence from the illuminated layer is proportional to the intensity of the radiation examined by using the energy of the focus spot on the corresponding layers, the zeroth-order beam is checked on the substrate plane and the first-order beam is described on the plane of the recording layer. The cross sections of the point spread functions (PSF) for the two beams are shown in Figure 2 to describe the intensity of the radiation. The PSF of the first-order beam is wider than that of the zeroth-order beam; however, the PSFs are centrally peaked with a clearly defined first Airy ring.

### 2.3. DOE Design

**Figure 3.**
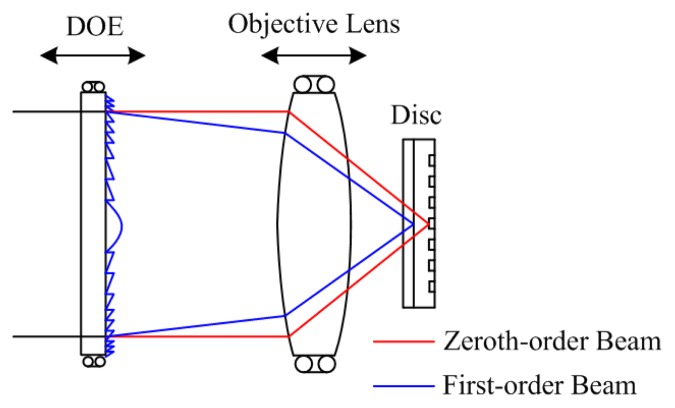
Beam separation by the DOE.

The DOE is a key component of this system; it is used as a beam separation device, as shown in Figure 3. As stated above, the optical power of the DOE's diffractive surface is designed as zero. The DOE is designed in the optical model to fulfill the requirement of the diffractive angle and the diffraction efficiency.

In the optical design, we obtain the phase function of the DOE as follows:

(1) $$\Phi (\rho )=A_{1}\rho^{2}+A_{2}\rho^{4}=-353.816\rho^{2}+0.461\rho^{4}$$

where *ρ* is the normalized polar coordinate of the surface, and *A*_1_ and *A*_2_ are coefficients. Using the present manufacturing technology, it is difficult to fabricate a surface which absolutely matches with the phase function, thus, the surface is processed in a discrete way and divided into annular diffractive zones of different radii. The phase at the edge of each zone is the integral multiple of 2π, as the phase differs exactly by 2π from adjacent zones. Therefore, we can determine the radius of each zone. The results show that there are 56 diffractive zones with a minimum grating period of 13.4 μm.

The diffraction efficiency of each order of the diffracted beam varies according to the DOE depth. The first-order beam can be maximized when the depth is calculated by Equation (2) [12]:

(2) $$h_{\text{max efficiency}}=\lambda /(n-1)$$

where *λ* = 0.78 μm is the wavelength, *n* = 1.519 (using the plastic material 480R from ZEON Co., Tokyo, Japan) is the refractive index of the DOE. Considering the irradiance requirements of the first-order beam and the zeroth-order beam, however, the depth h of the DOE is designed as 1.2 μm. According to Equation (3), the diffraction efficiency of the zeroth-order beam is 6.3% and that of the first-order beam is 85.8%, where *m* is the diffraction order [13]:

(3) $$\eta_{m}={\left[\sin\left\{\pi \left[m-\left(\frac{n-1}{\lambda }h\right)\right]\right\}/\left[\pi \left(m-\frac{n-1}{\lambda }h\right)\right]\right]}^{2}$$

The surface profile of the DOE is expressed as follows [14]:

(4) $$Z=h\left\{\frac{1}{2\pi }\Phi (\rho )+\mathrm{int}\left[\frac{1}{2\pi }\cdot \left|\Phi (\rho )\right|\right]\right\}$$

We obtain the surface profile of each diffraction zone *i* (*i* = 1, 2, …, 56) as follows:

(5) $$Z_{i}=C_{1}r^{2}+C_{2}r^{4}+k_{i}$$

where *r*(mm) is the actual polar coordinate of the surface, *C*_1_ = −0.030033 (mm^−1^) and *C*_2_ = 1.739151 × 10^−5^ (mm^−3^). The coefficient *k_i_* adopts different constants in different zones, as calculated by Equation (6):

(6) $$k_{i}=1.2\times 10^{-3}\cdot \mathrm{int}(\left|-25.027362r^{2}+1.449292\times 10^{-2}r^{4}\right|)\;(\text{mm})$$

## 3. Servo Simulation

### 3.1. Tracking Servo

Jitter and tilt are unavoidable while the disc is rotating. The tracking servo is necessary to maintain the proper focus position of the beam so that the beam is maintained on track to record and read data correctly. The push-pull method is used in the tracking servo, as the medium and the land/groove substrate in the system are separated from each other and there is no data point on the substrate.

**Figure 4.**
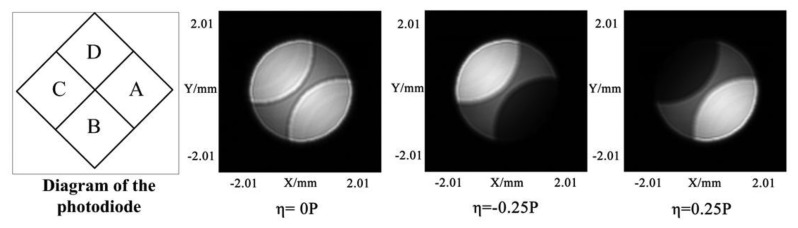
Light intensity distribution of the reflected beam with different TP deviation. η is the TP deviation. P is the track pitch.

The push-pull method is based on the interference effect, as the disc with grooves can be viewed as a reflection grating [15]. The light intensity distribution of the servo beam reflected by the land/groove substrate is calculated using the scalar diffraction theory [16]. Figure 4 shows the light intensity distribution of the reflected beam with different track pitch (TP) deviation, where the light intensity on the left side and right side of the beam is not equal. The diagram of the photodiode in the system is also described in Figure 4.

**Figure 5.**
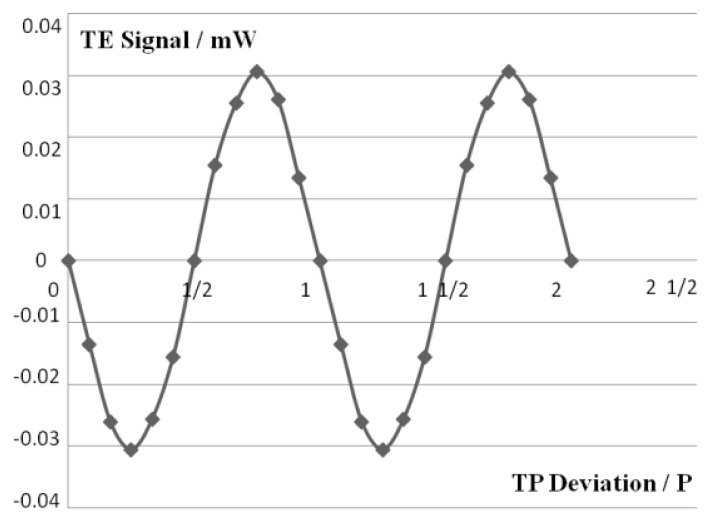
Tracking error signal. The x-axis represents normalized TP deviation by track pitch P.

The tracking error (TE = (I_C_ + I_D_) − (I_A_ + I_B_), I_A_, I_B_, I_C_ and I_D_ represent the light intensity distribution on the corresponding quadrant) signal is obtained by the use of the photodiode. This curve in Figure 5 is similar to the typical TE curve [17], shows that the tracking servo can perform well.

### 3.2. Focusing Servo

The distance between the objective lens and the disc should be constant to keep focus. However, the distance may change because of the disc jitter and tilt. A focusing servo in the servo module is used to maintain a constant distance.

**Figure 6.**
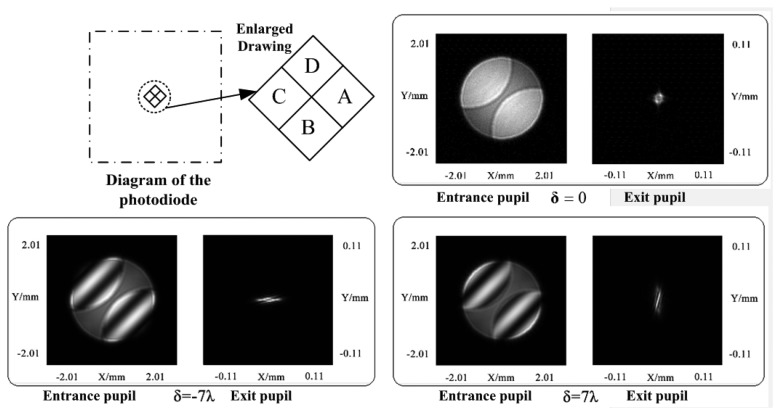
Light intensity distribution of the reflected beam at the entrance pupil (left one in subplot) of spherical lens and the exit pupil (right one in subplot) of cylindrical lens with a different defocus distance. δ is the defocus distance. *λ* is the wavelength.

The astigmatic method is performed in the focusing servo by a cylindrical lens [18]. Similarly, we calculate the light intensity distribution of the servo beam reflected by the land/groove substrate using the scalar diffraction theory. Figure 6 shows the light intensity distribution of the reflected beam at the entrance pupil of a spherical lens and the exit pupil of a cylindrical lens with different defocus distances. It corresponds well with the astigmatic method principle.

**Figure 7.**
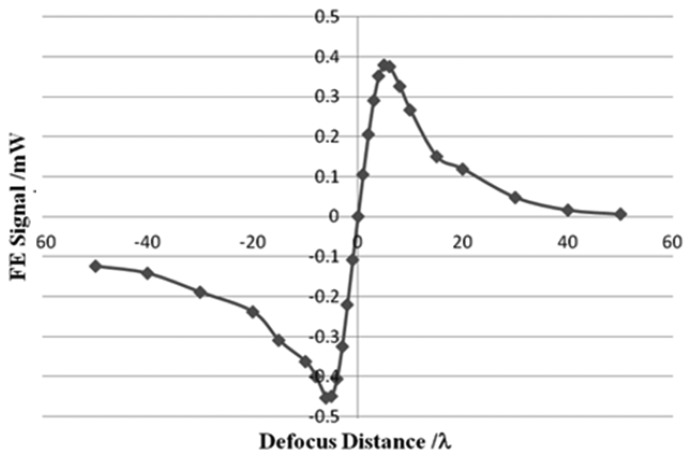
Focusing error signal. The x-axis is normalized defocus distance by wavelength.

The focusing error (FE = (I_B_ + I_D_) − (I_A_ + I_C_)) signal is also obtained by the use of the photodiode. The FE curve shown in Figure 7 has linear regions and nonlinear regions, similar to a typical S-curve [19]. The linear region is in the range of 8 λ, which approaches that of a DVD system. The length of the linear region is 6.24 μm and the sensitivity is 0.099 mW/*λ*. Because of the linear region, the focusing servo can perform accurately.

### 3.3. Layer Selection

**Figure 8.**
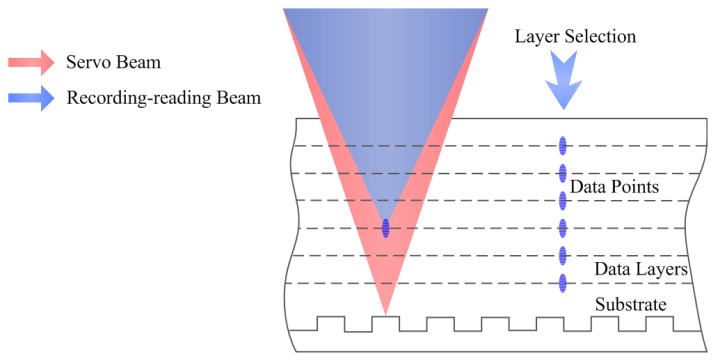
Layer-selection process.

**Figure 9.**
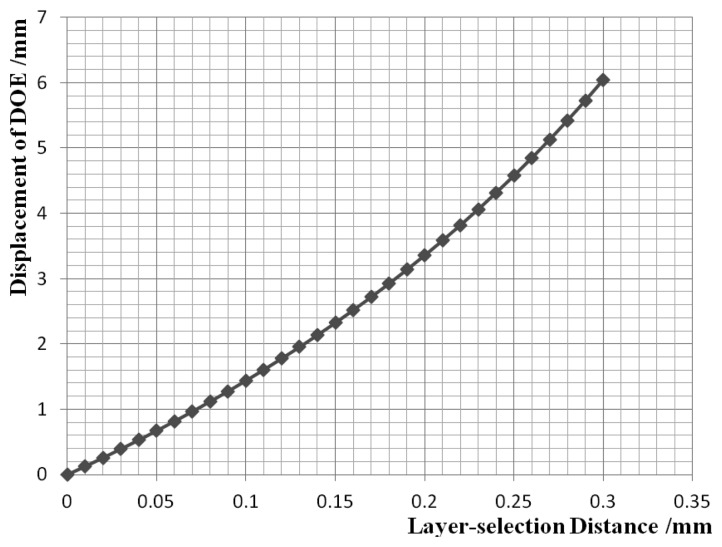
Layer-selection distance *versus* displacement of DOE.

The layer-selection process is shown in Figure 8, where the recording layer is selected by moving the DOE so that multilayer recording and reading are possible. At the same time, the servo beam is focused on the land/groove substrate, regardless of any DOE movement. The layer-selection distance *versus* displacement of DOE is shown in Figure 9. There is an almost linear correlation between the displacement of the DOE and the layer-selection distance. As a result, the layer-selection process could be controlled easily. When the layer-selection distance ranges from zero to 0.3 mm, the displacement of DOE is in the range of 0 to 6.045 mm. The displacement of the DOE is small, so it is possible for the system to be compact and miniaturized.

## 4. Optical Experiment

**Figure 10.**
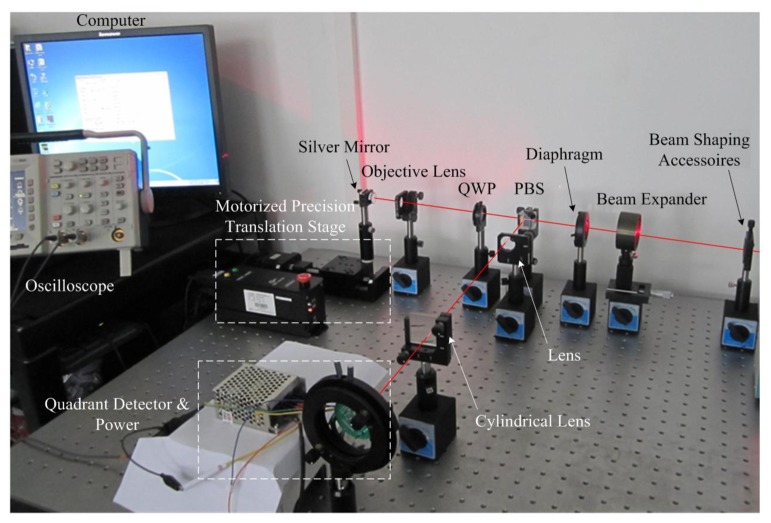
Experimental platform of light path for focusing servo beam.

The experimental platform of the light path for the servo beam is shown in Figure 10. In the dual-layer recording [6], the femtosecond pulsed laser beam at the wavelength of 780 nm with a repetition rate of 82 MHz was employed as the excitation source. In the current experiment, we use the laser at the wavelength of 780 nm with 5 mW average power instead to simplify the opto-electronic platform as the characteristics of wavelength and polarization are not investigated. It uses an objective of NA = 0.6.

### 4.1. Focusing Servo Experiment

Focusing servo experimental platform is shown in Figure 10. The beam is shaped and collimated. After passing through the diaphragm, the parallel beam is focused on a silver mirror by the objective lens. The reflected beam is separated from the main optical path by a PBS. Then the beam is focused on a CCD after passing through a spherical lens and a cylindrical lens. The spot pattern is transmitted to a computer by the CCD, and the FE signal is detected by a quadrant detector. The mirror, serving as a reflective surface on the disc, is assembled on a motorized precision translation stage with a resolution of 0.001 mm. The states of the disc such as in-focus and defocus are simulated by controlling the movement of the translation stage. The response of the quadrant detector is 0.53 A/W when the wavelength is 780 nm, according to the spectral response.

**Figure 11.**
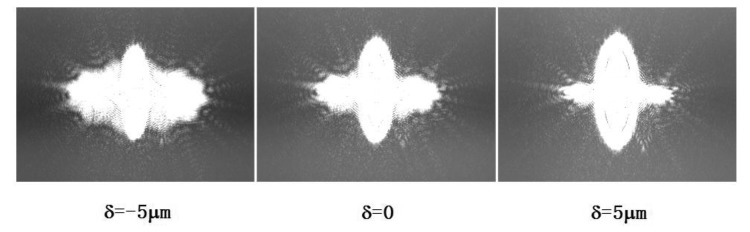
Spot pattern at different defocus distances.

The spot pattern obtained by the CCD is shown in Figure 11. When the mirror is placed in different locations, the resulting spot pattern is varied. The spot pattern is rotationally symmetric; and the FE signal is 0 when the defocus distance δ = 0.

When δ = −5 μm, the spot pattern is nearly elliptical with a horizontal major axis and a FE signal of less than 0. When δ = 5 μm, the spot pattern is nearly elliptical with a vertical major axis and the FE signal is greater than 0. These experiment phenomena correspond well with the simulation results.

**Figure 12.**
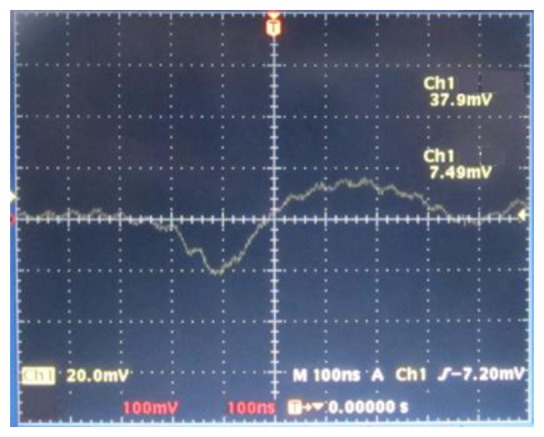
The waveform of the FE signal on the oscilloscope.

We used an oscilloscope to observe the output signal of the quadrant detector when the translation stage is moving cross the focal plane at a speed of 32 mm/s. The obtained waveform is shown in Figure 12. It is thus clear that the waveform is similar to a typical S-curve. There is linear region near the focal plane, and the FE signal is nearly 0 on both ends, far away from the focal plane.

The length of linear region is:

(7) $$s=v\cdot t=32\times 10^{3}\times 0.22\times 10^{-3}=7.04(\mu m)$$

The relative error of linear region is:

(8) $$\gamma_{s}=\frac{\left|7.04-6.24\right|}{6.24}\times 100\%=13\%$$

The sensitivity of linear region is:

(9) $$k=\frac{V\cdot 10^{3}}{10^{2}\cdot \eta \cdot s/\lambda }=\frac{37.9\times 10^{-3}\times 10^{3}}{10^{2}\times 0.53\times 7.04/0.78}=0.08(mW/\lambda )$$

The relative error of sensitivity is:

(10) $$\gamma_{k}=\frac{\left|0.08-0.099\right|}{0.099}\times 100\%=19\%$$

Thus the values are simplified as *s* = (7±1)*μm*, *k* = (0.08±0.02)*mW* / *λ*. There are some errors between the experimental results and the simulation results as the waveform is not smooth; however, the errors are within certain limits. One of the main reasons for the errors is the imprecise positions of elements. The vibration of the translation stage will also contribute to the errors.

### 4.2. Layer Selection Experiment

**Figure 13.**
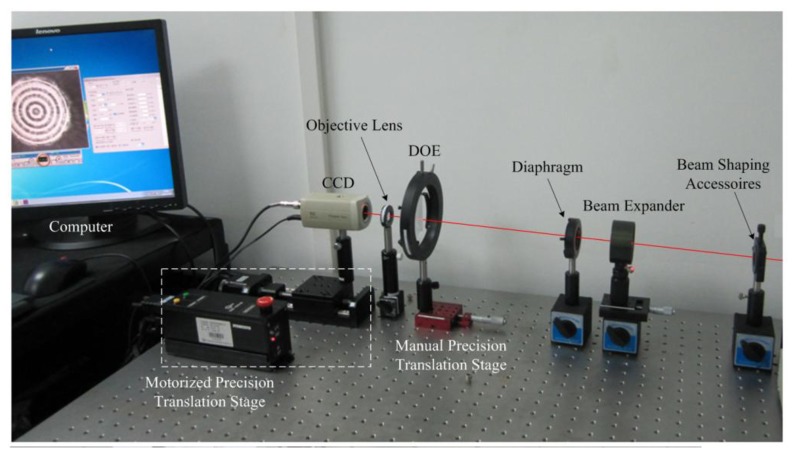
Experiment platform of light path for recording-reading beam.

Figure 13 shows the layer selection optical experiment. After passing through a diaphragm, a DOE, and an objective lens, the parallel beam is focused on a CCD. The spot pattern is transmitted to a computer by the CCD. The DOE is assembled on a manual precision translation stage with a resolution of 0.005 mm, and the CCD is assembled on a motorized precision translation stage with a resolution of 0.001 mm. The layer-selection process is simulated and the properties of the DOE are verified by controlling the movement of the DOE. However, effects observed in real recording layer should be discussed in the further studies as the experiment of layer selection is employed without real layer.

**Figure 14.**
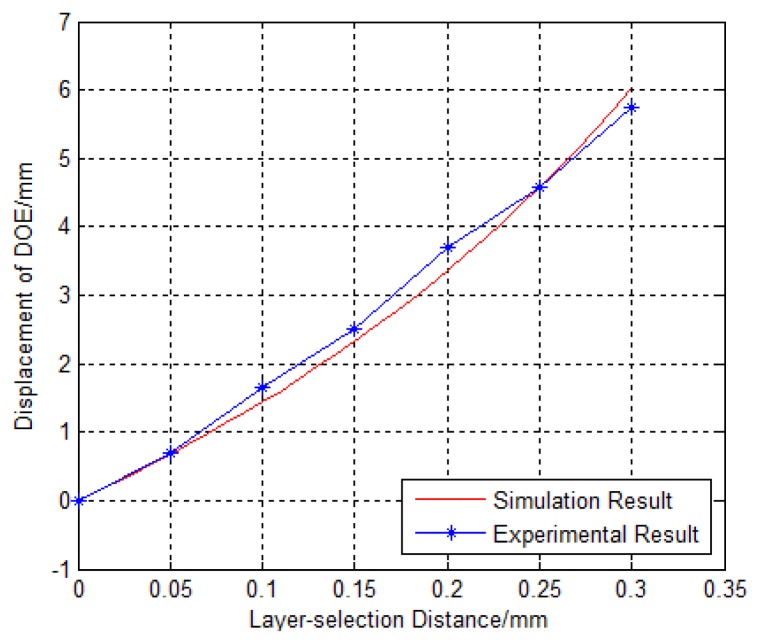
Results of the layer-selection distance *versus* displacement of DOE.

The motorized precision translation stage is controlled to move with a step of 50 μm. Every time it is moved, the manual precision translation stage is moved in the same direction to keep the beam converging. The measured relationship between the layer-selection distance and the displacement of the DOE is shown in Figure 14.

The results agree with the simulation results. The maximum absolute error between the simulation and the experiment results is:

(11) |e|=|3.691−3.356|=0.335(mm)

and the maximum relative error is:

(12) $$\gamma =\frac{\left|1.652-1.439\right|}{1.439}\times 100\%=15\%$$

## 5. Conclusions

In this paper, we have proposed a compact single laser opto-electronic system layout applied for a novel optical information storage sensing system by the use of a diffractive optical element. The design results are described both in a simulation and experimentally. The DOE is used to separate the recording-reading beam from the servo beam in a single optical path with one objective lens. The DOE is also designed to control the layer-selection process; and there is a linear correlation between the displacement of DOE and that of the layer-selection. The minimum grating period of the DOE is 13.4 μm, and the depth of the DOE is 1.2 μm, which makes it capable of being fabricated. The system has been designed to possess a linear region of 8 λ which is compatible with the current DVD servo module. The length of the linear region is 6.24 μm and the sensitivity is 0.099 mW/*λ*. The proposed system is available for LSPR detection of gold nanorods in a three-dimensional space, which is feasible for larger storage capacity in a given range of layer thickness. The system can also be applied for optical information sensing in micromachining, optical encryption and some relative sensors.
